# Eliciting interval beliefs: An experimental study

**DOI:** 10.1371/journal.pone.0175163

**Published:** 2017-04-05

**Authors:** Ronald Peeters, Leonard Wolk

**Affiliations:** 1 Department of Economics, School of Business and Economics, Maastricht University, Maastricht, The Netherlands; 2 Department of Finance, Vrije Universiteit Amsterdam, The Netherlands; Universidad de Alicante, ITALY

## Abstract

In this paper we study the interval scoring rule as a mechanism to elicit subjective beliefs under varying degrees of uncertainty. In our experiment, subjects forecast the termination time of a time series to be generated from a given but unknown stochastic process. Subjects gradually learn more about the underlying process over time and hence the true distribution over termination times. We conduct two treatments, one with a high and one with a low volatility process. We find that elicited intervals are better when subjects are facing a low volatility process. In this treatment, participants learn to position their intervals almost optimally over the course of the experiment. This is in contrast with the high volatility treatment, where subjects, over the course of the experiment, learn to optimize the location of their intervals but fail to provide the optimal length.

## Introduction

Schlag and van der Weele [1, 2] propose the interval scoring rule as a mechanism to elicit subjective beliefs when these beliefs involve a distribution over a continuum of events. One interesting property of this scoring rule is that in case the distribution reflecting the individual’s subjective belief is single-peaked, it is incentive compatible for this individual to construct an interval that contains the mode of this distribution. Several papers have implemented variations of the interval scoring rule [3–6], yet no paper exists that evaluates individual behavior using this elicitation mechanism.

In this paper we investigate the intervals individuals report when being incentivized via the interval scoring rule. We consider the quality of their choices taking the risk-neutral optimal interval as the benchmark, whether the mode is contained in the constructed intervals, and whether individuals update their intervals in a manner consistent with the directional learning hypothesis [7, 8]. Furthermore, we investigate whether sub-optimal choices are due to the location or length of the chosen interval. Finally, since individuals are known to be slow learners in noisier environments [9], we study how elicited intervals relate to the degree of underlying uncertainty.

To accomplish our aim, we design an experiment where subjects predict the termination time of a time series that is to be generated from a fixed but, to the subjects, unknown random process. Participants state their beliefs about the next termination time using the interval scoring rule and the task is repeated over a sequence of twenty periods. We implement two treatments: one in which the stochastic process exhibits a relatively low variance and one where it exhibits a relatively high variance.

Central to our design is that we allow subjects to learn from different sources of information: subjects do not only observe the termination time of the time series but also, graphically, the path it took to that termination time. Our design, thereby, creates an environment in which subjects learn from information that is known precisely (termination times) on the one hand and information that requires subjective judgments (path to the termination point) on the other hand.

We find that elicited intervals are significantly better in the low volatility treatment than in the high volatility treatment. In both treatments, individuals improve their performance over time, although in the high volatility treatment they mainly improve the choice of location given the length, but fail to choose the correct length. Interestingly, behavior in the experiment does not appear to be significantly affected by risk preferences. This is in line with Harrison et al. [10], who show that, under the assumption of expected utility theory, when eliciting subjective beliefs over continuous events, one does not need to correct those beliefs for the subject’s risk preferences. Yet, interestingly, this is in contrast with beliefs elicited over binary outcomes which are affected by an individual’s risk tolerance (see for instance [11]) and when individuals follow rank dependent utility theory [12].

Important to note is that the probability distribution of hitting times is unknown to the participants in our experiment. As a consequence, decisions are not made under risk, but rather under uncertainty. Therefore, it is possible that decisions are influenced by preferences for ambiguity. In Trautmann and Zeckhauser [13], individuals choose between alternatives that have known and unknown probabilities. The authors conduct a repeated experiment where it is beneficial to learn about the unknown probability, but show that subjects shun away from uncertain choices and thereby forgo valuable learning opportunities. Our experiment differs significantly from theirs since we do not allow for self-selection into the two different environments (high and low volatility) that we study. Instead, the subjects are aware of the fact that the process from which random draws are generated is fixed over the course of their experiment and we thereby do not allow them to shun away from the uncertainty that they face. Consequently, subjects are required to respond to the uncertainty via the design of their intervals.

## Materials and methods

All experiments were conducted with the informed consent of healthy adult subjects who were free to withdraw from participation at any time. Only individuals who voluntarily entered the experiment recruiting database were invited, and informed consent was indicated by electronic acceptance of an invitation to attend an experimental session. The experiments were conducted following the peer-approved procedures established by Maastricht University’s Behavioral and Experimental Economics Laboratory (BEElab). Our study was approved by the BEElab at a public ethics review and project proposal meeting that is mandatory for all scholars wishing to use the BEElab facilities.

**Fig 1:**
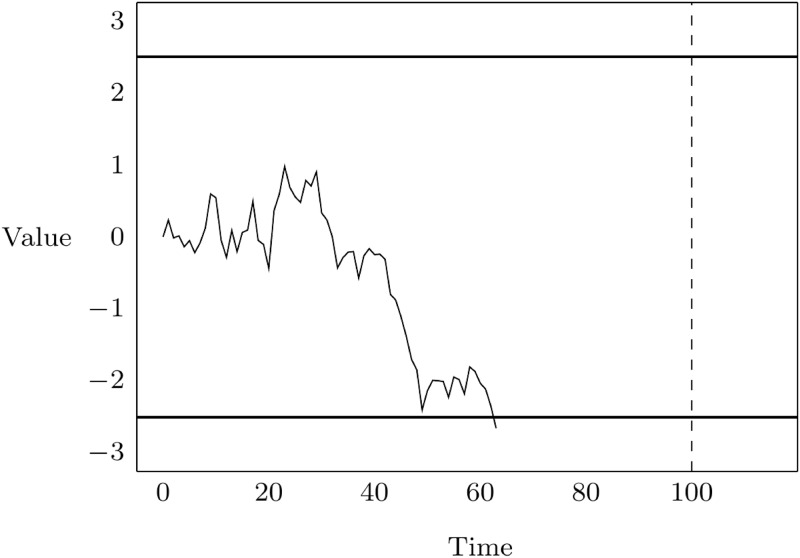
An example of a time series.

In the experiment subjects are exposed to a random process that starts at a value of zero at time *t* = 0 and runs from there in discrete time-steps. At each unit of time the value is incremented with a real number (possibly negative) that is drawn randomly according to a normal distribution with mean zero (hence, there is no drift) and a fixed but to the participants unknown variance. The process terminates either when the value crosses the lower boundary at −2.5, crosses the upper boundary at +2.5, or has reached time *t* = 100 without having reached one of these boundaries. Fig 1 shows one time series generated by this process that led to a termination at the lower bound at time *t* = 63.

In a sequence of twenty rounds, the task of the participants in this experiment is to predict the termination time of the upcoming time series. Participants were not informed about the details of the underlying random process, but knew that it was kept constant throughout the experiment. In the course of twenty rounds of decision making, the participants gradually learn about the stochastic process, possibly giving rise to a gradual improvement in their predictions.

Prior to the first round, participants saw one realized time series in the instructions (by means of a graph like Fig 1) and were shown one animation of a randomly generated time series on screen. Next, they were asked to indicate the time interval in which they believe the next time series is going to hit one of the boundaries, conditional on the time series to terminate before time *t* = 100. The decision was made by positioning two triangular cursors along the time line between *t* = 0 and *t* = 100. Participants were incentivized by means of an interval scoring rule [1]: a participant expressing the belief that, conditional on the time series to terminate before time *t* = 100, it to hit one of the boundaries within the time interval $\left[\hat{x},\hat{y}\right]$ received $100\cdot {\left(1-\frac{\hat{y}-\hat{x}}{100}\right)}^{2}$ ECU (Experimental Currency Units) if the time series indeed terminated within the given time interval and received nothing otherwise. The payoff that could potentially be obtained is larger when a smaller interval is selected and the potential payoff was shown on-screen in real-time while cursors were moved along the time line. After having confirmed their predictions, participants were shown the animation of the time series that was generated for the first round, whereafter the task was repeated in the second round. This procedure continued until the last (twentieth) round.

Finally, the participants participated in a short cognition task in which we elicited their perceptual reasoning ability, their risk attitude, and a few personal characteristics, including gender and age. For the cognition task, we used the symbol-digit correspondence test from the Wechsler Adult Intelligence Scale (WAIS), in which subjects had 90 seconds to find as many correspondences between symbols and numbers as they could, using the correct number for each symbol. The speed and accuracy of this task under time pressure determine an individual’s perceptual reasoning ability (cf. [14]). Risk attitude was elicited by the direct approach as suggested in [15].

A random selection of subjects from our subject pool (mainly students in business and economics) were invited to participate in an economic experiment via ORSEE [16]. The sessions were run in the BEElab at Maastricht University in September 2013 and March 2016. The instructions were paper-based and the prediction phase was computerized using z-Tree [17]. In total 72 students participated: half of them participated in the low volatility treatment with the standard deviation of the normal distribution being equal to 0.1885, the other half participated in the high volatility with this standard deviation being set at 0.2270. These standard deviations are chosen such that the probability of the process to terminate before *t* = 100 equals approximately 1/3 in the low volatility treatment and 2/3 in the high volatility treatment. All participants in a treatment were shown the same animations (graph in instructions, on-screen animation before first decision, and all other twenty animations), in the same order, and the series of time series were generated by a statistical software package and were not subject to experimental manipulation. At the end of the session, in order to limit excessive variance in earnings while keeping saliency of incentives for each decision, for each participant individually, eight random draws (with replacement) over the payoffs that were earned in the twenty rounds were made. The final earnings of the participants consisted of the amount of ECUs collected in these eight tasks exchanged into Euros at a conversion rate of 6 Eurocents for each ECU and a 3 Euro show-up fee. Each experimental session lasted about 60 minutes and the average earnings of the subjects was 16.59 Euro. All instructions, software, data files and codes used for analysis are retrievable from Figshare (doi: 10.6084/m9.figshare.3997203; url: figshare.com/s/424cc9f37bfe52f0be75).

**Fig 2:**
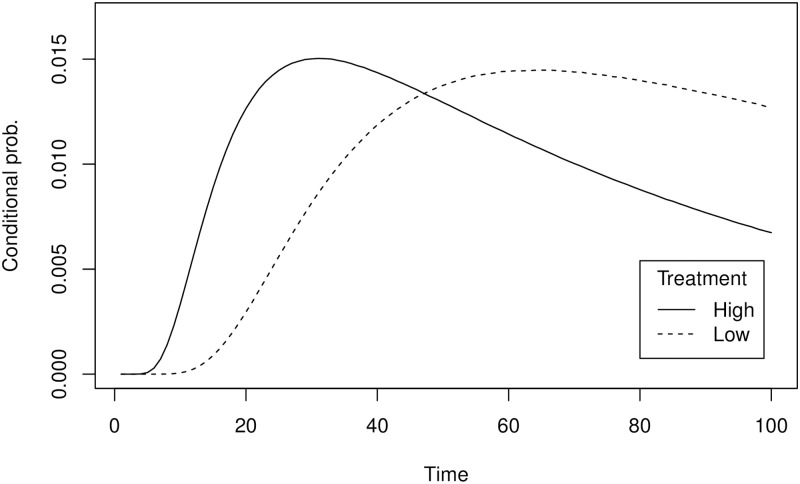
Distribution over termination times conditional on termination before *t* = 100. The dashed curves relate to the low volatility treatment; the solid curve to the high volatility treatment.

Fig 2 presents the true distribution over termination times, conditional on termination before *t* = 100, for the two treatments. The mode of this distribution is at 66 for the low volatility treatment and at 31 for the high volatility treatment. Given the incentives provided, when having perfect knowledge of this true distribution, a risk neutral individual maximizes her expected payoff by choosing the interval [51, 83] in the low volatility treatment and the interval [21, 51] in the high volatility treatment.

One advantage of the use of the time series is that participants collect more information in one round of decision making as in comparison to the classical urn experiments. In fact, already before making the first decision, participants can form a good impression of the process. S1 Fig shows the densities of the innovations on the basis of the innovations giving rise to the figure in the instructions and the animation they saw prior to the first decision together with the true normal distributions, and S2 Fig shows the densities over termination times based on these innovations together with the true distribution. The simulations are based on random draws with replacement of the innovations plotted in S1 Fig and are constructed using one million replications.

## Results

In Table 1 we present the summary statistics of our experiment. The upper part shows the summary statistics of the main characteristics of the participants in our experimental sessions. The ratio of males was slightly larger in the low volatility treatment; so was the number of correctly identified symbols in the cognition task. There are no substantial differences in age and risk attitudes (where the value 0 indicates extreme risk aversion and the value 10 extreme risk loving) between the participants in the two treatments.

**Table 1:** Summary statistics of the participants in the experiment.

	Mean value (std.dev)					
	All		Low		High	
Age (years)	21.0	(2.2)	20.8	(1.8)	21.1	(2.6)
Gender (%, Male = 1)	54.2		58.3		50.0	
Risk attitude (0–10)	6.3	(1.9)	6.1	(1.9)	6.6	(1.8)
Cognitive ability (number)	40.1	(6.2)	40.6	(6.6)	39.6	(5.8)
Lower bound (0–100)			42.8	(15.2)	32.3	(18.4)
Upper bound (0–100)			80.0	(15.7)	75.8	(17.8)
Length			38.2	(14.3)	44.5	(17.7)
Location (mid-point)			61.4	(13.7)	54.1	(15.8)
Exp. payment (in ECU)			17.8	(4.3)	14.5	(4.5)

The lower part of this table shows the average intervals constructed and the average expected payment, where averages are taken over all individuals over all twenty periods and the expectation is based on the expected payment given the interval chosen on the basis of the true distribution. The standard deviation of interval length and location is mainly due to variations across subjects rather than due to variations within subjects over time. The one-period auto-correlation of length (location) is 0.6459 (0.3444) and 0.6439 (0.3028) in the low and high volatility treatments respectively.

The average interval in the low volatility treatment almost fully captures the interval that a risk neutral individual would optimally choose (when knowing the true distribution) and the mode of the true distribution. In the high volatility treatment a substantial part of the risk neutral optimal interval is not captured in the average interval chosen; even the mode of the true distribution is just missed by the average interval. In both treatments subjects design longer intervals than a risk neutral individual would optimally do. The mis-positioning of the intervals in the high volatility treatment relative to the low volatility treatment leads to subjects’ expected payment being significantly higher in the low volatility treatment compared to the high volatility treatment (Mann-Whitney U: *p* < 0.001).

### Choices

**Fig 3:**
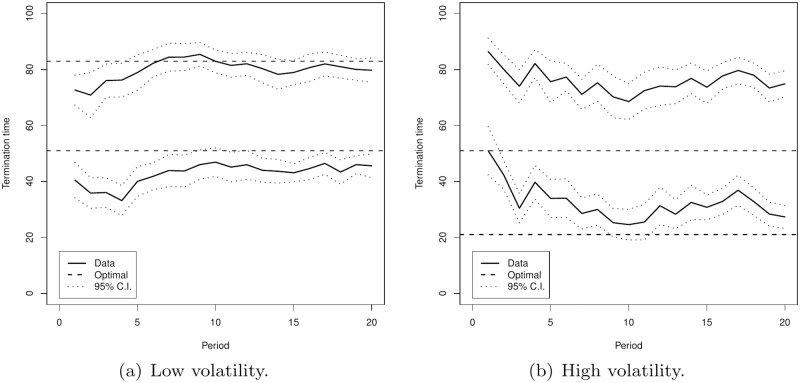
The solid lines indicate the average intervals (identified by the average lower an upper bounds) over time. The dashed lines indicate the optimal intervals for a risk neutral individual and the dotted lines indicate the 95% confidence interval around the average upper and lower interval bounds.

Panel (a) of Fig 3 presents the evolution of the average interval (identified by the average lower an upper bounds) chosen during the course of the experiment for the low volatility treatment, while Panel (b) shows those of the high volatility treatment. The dashed lines indicate the risk-neutral optimal interval. We see that there is some learning in the first periods and on average behavior stabilizes in the low volatility treatment while this is less so in the high volatility treatment. While subjects in the low volatility treatment appear to adjust their bounds toward the optimal interval, there appears to be persistent mis-positioning of the upper bound in the high volatility treatment. The earlier observed properties on the positioning of the intervals, relative to the risk-neutral optimal intervals and the lengths of the intervals appear not to be an artefact of averaging over rounds but a persistent property. The risk-neutral optimal intervals have the property that the upper bound of the interval in the high volatility treatment should be equal to the lower bound of the interval in the low volatility treatment. Averaged over time the former is 33 points above the latter and there is no time period in which these bounds differ by less than 21.7. The regression results presented in Table 2 indicate that over time the intervals marginally shrink in the low volatility treatment and marginally expand in the high volatility treatment. According to S1 Table this is due to a significant increase (decrease) in the lower bound of the intervals while the simultaneous increase (decrease) in the upper bound is smaller and not significant.

**Table 2:** Interval length and whether the mode is contained in the interval against individual characteristics.

	Interval length		Mode contained (marginal effect)	
Treatment	Low	High	Low	High
Constant	55.5813 (44.2399)	-134.6241 * (77.3593)		
2nd Half	-2.9611 ** (1.2229)	2.6917 ** (0.9962)	0.0339 * (0.0182)	0.1355 *** (0.0310)
Gender	-0.8061 (3.6427)	6.4250 (4.8765)	0.0713 * (0.0428)	0.0233 (0.0489)
Risk attitude	-0.2472 (0.9709)	-0.6217 (0.8495)	-0.0053 (0.0076)	-0.0090 (0.0185)
Cognitive ability	-0.6075 (2.1555)	9.1181 ** (4.0097)	-0.0708 (0.0561)	0.0753 (0.0518)
Cognitive ability (squared)	0.0063 (0.0267)	-0.1140 ** (0.0514)	0.0009 (0.0007)	-0.0009 (0.0007)
Observations	720	720	720	720
R-squared	0.0149	0.0878	0.0945	0.0401

Standard errors clustered on the individual level in parentheses.

*** *p* < 0.01,

** *p* < 0.05,

* *p* < 0.1.

One property of the interval scoring rule is that if a subject’s belief distribution over termination times is single-peaked, and this peak is unique, then the mode of this distribution should be contained in the reported interval [1]. We see that for the low volatility treatment the mode of the true distribution (at 66) is contained in the average interval chosen during the whole course of the experiment; for the high volatility treatment, the true mode (at 31) is contained in the average interval only in half of the periods.

**Fig 4:**
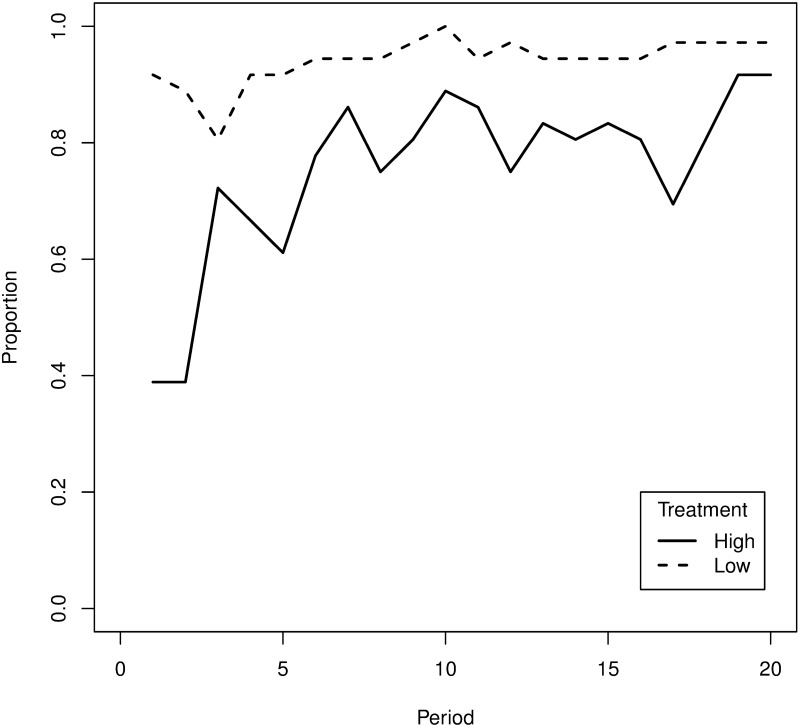
Share of intervals containing the true mode.

Due to the flatness of the true distributions at the mode, it is hard for subjects to learn or to identify the true mode. In fact, millions of simulations are required to numerically identify the true mode. It is therefore not to be expected that our experimental subjects would be able to learn to do so within twenty rounds (even when taking into account that during one round they learn more about the process than just the termination time). Allowing for a certain degree of mis-identification, Fig 4 shows the share of intervals that contained the true mode at each time period. We classify each interval, that intersects with a termination time that is at least 95% as likely to realize as the true mode, as containing the true mode. This implies that the range of values that could be considered as modes are [51, 84] in the low volatility treatment and [25, 40] in the high volatility treatment; though, not allowing for mis-identification (i.e. only accepting the true mode) does not have any impact on the main findings. The figure shows that after the first five periods, at least 29 of the 36 subjects had an approximate mode contained in their interval in the low volatility treatment, while this was the case for at most 26 of the 36 subjects in the high volatility treatment. The fraction of subjects in the high volatility treatment that make a good forecast in this respect is never above the fraction of intervals containing the approximate mode in the low volatility treatment.

The last two columns of Table 2 reveal how interval lengths chosen and whether or not intervals contain the true mode relate to individual characteristics. Gender and risk attitude do not have a significant effect on the choice of interval length. Also a Mann-Whitney U test comparing the chosen interval lengths in the last ten rounds between the one-thirds of the subjects that are most and least risk averse did not indicate a significant difference (Low: *p* = 1.0000; High: *p* = 0.3451). The same holds for the first ten rounds (Low: *p* = 0.7938; High: *p* = 0.3824). Cognitive ability has no effect on the chosen interval length in the low volatility treatment, but shows a quadratic relation in the high volatility treatment with low and high cognitive skilled choosing smaller intervals compared to the middle group. None of the individual characteristics is strongly predictive for the mode being contained in the interval.

### Performance

**Fig 5:**
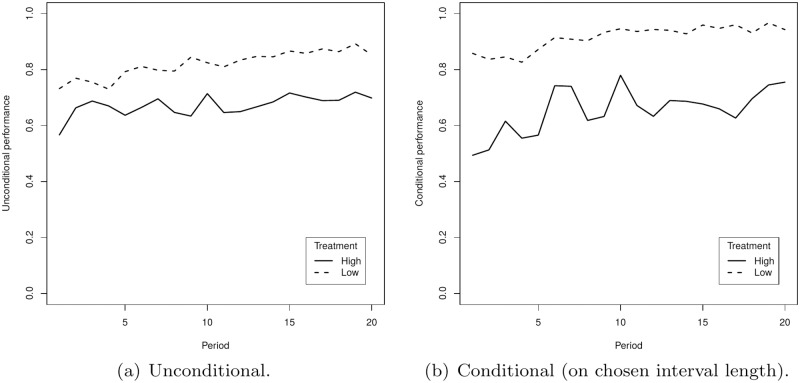
Performance over time in the low volatility (dashed) and the high volatility (solid) treatment.

In each treatment we measure individual performance in the prediction task using two different methods. The ‘unconditional’ performance measure captures the expected payoff relative to the maximum expected payoff that can be obtained in the respective treatment had the true distribution been known and is given by *π*/*π*^max^ where *π* is the expected payoff and *π*^max^ the maximum possible payoff. The ‘conditional’ performance measure conditions the performance on the chosen interval length and equals $\left(\pi -\pi_{\ell }^{\min}\right)/\left(\pi_{\ell }^{\max}-\pi_{\ell }^{\min}\right)$ where *π* is the expected payoff and $\pi_{\ell }^{\min}$ and $\pi_{\ell }^{\max}$ are the minimum and the maximum possible payoff conditional on the chosen interval length. Fig 5 shows the development of the average individual performance according these two measures for the two treatments. In general, performance improves over time and appears to be better in the treatment with the less volatile process according to both measures.

Table 3 presents for both treatments the result of cross-sectional regressions of the individual performance (for both measures) on the participants’ individual characteristics. In order to control for general learning, we included the variable ‘2nd half’ as indicator for the last 10 rounds; this variable is positive and significant, suggesting that subjects learn to forecast the underlying process better over time. Gender and risk attitude do not have a significant effect on performance. Also a Mann-Whitney U test comparing individual performance in the last ten rounds between the one-thirds of the subjects that are most and least risk averse did not indicate a significant difference, neither for the unconditional performance (Low: *p* = 0.9479; High: *p* = 0.5079) nor for the conditional performance (Low: *p* = 0.3237; High: *p* = 0.4639). The same holds for the first ten rounds, for both unconditional (Low: *p* = 0.3575; High: *p* = 0.9723) and conditional performance (Low: *p* = 0.2921; High: *p* = 1.0000). Cognitive ability has no effect on individual performance in the high volatility treatment, but we find a significant non-linear effect in the low volatility treatment (for both measures of performance): individuals with low and high cognitive ability scores perform better than those with intermediate scores.

**Table 3:** Individual performance.

	Low volatility		High volatility	
	Unconditional	Conditional	Unconditional	Conditional
Constant	1.7912 *** (0.3754)	1.6674 *** (0.2826)	1.9678 * (1.1504)	-0.5089 (0.7788)
2nd Half	0.0692 *** (0.0146)	0.0611 *** (0.0137)	0.0283 * (0.0149)	0.0586 *** (0.0210)
Gender	0.0693 * (0.0371)	0.0392 (0.0295)	-0.0188 (0.0595)	0.0204 (0.0537)
Risk attitude	-0.0108 (0.0076)	-0.0064 (0.0061)	-0.0137 (0.0084)	0.0081 (0.0143)
Cognitive ability	-0.0518 ** (0.0195)	-0.0402 ** (0.0153)	-0.0644 (0.0565)	0.0503 (0.0390)
Cognitive ability (squared)	0.0007 *** (0.0002)	0.0005 ** (0.0002)	0.0008 (0.0007)	-0.0006 (0.0005)
Observations	720	720	720	714
R-squared	0.0996	0.0693	0.0439	0.0260

Standard errors clustered on the individual level in parentheses.

*** *p* < 0.01,

** *p* < 0.05,

* *p* < 0.1.

**Fig 6:**
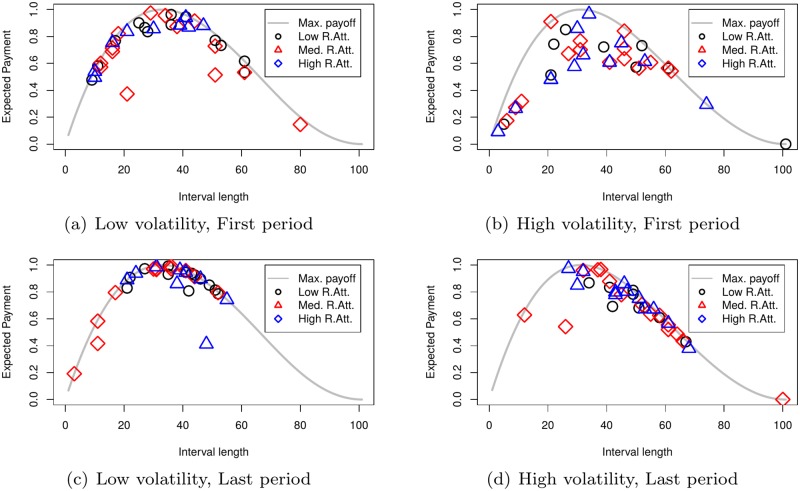
Individual performance against interval length for the two treatments in the first and last period.

It is somewhat surprising to see that risk attitudes neither affect interval length (Table 2) nor forecasting performance (Table 3). Fig 6 displays individual performance (*y*-axis) conditional on interval length (*x*-axis) for the low and high volatility treatments in the first and last period of decision making. Panels (a) and (b) show first period choices for the low and high volatility treatments respectively, while panels (c) and (d) show the same individuals’ choices in the last period. The curves in the plots identify the (normalized) maximum attainable expected payoff as a function of the chosen interval length. Three different geometric shapes are used to distinguish individuals from three different risk groups where, for each treatment, we split the subjects at the one-thirds and two-thirds quantile of their reported scores. In the figure, the circles refer to the individuals with the lowest risk tolerance, the triangles to those with medium risk tolerance, and the diamonds to those with the highest risk tolerance.

Comparing the performance in the first and last period, we see that the figure nicely illustrates the effects observed in Table 3. In the low volatility treatment, with the geometric shapes being close to the curves in both panel (a) and (c), subjects succeed to choose the location close to optimal given the chosen interval length already in the first period and still do so in the last period. Though, comparing the distribution of interval lengths over these two panels, we see that over time subjects improve in their choice of interval length (while they keep choosing the right location given the length). Moreover, there is no apparent difference in the distribution of interval lengths across risk groups (which we saw already in Table 2).

In the high volatility treatment, we do not observe the same effect (panel (b) and (d)). First, subjects do not succeed to choose the best location given the chosen interval length in the first period, but learn to do so over time. Second, while similar to the low volatility treatment the dispersion of interval lengths is reduced over time, we see that they cluster on a suboptimal level: subjects opt for too long intervals. Overall, this explains the lack of improvement in individual performance over time in this treatment. Again, there is no apparent difference in the distribution of interval lengths across risk groups.

### Dynamics of choices

Each period, after having chosen their interval, subjects immediately receive feedback on their choice. In this section we analyze the dynamics of subjects’ choices on the principle of directional learning [7, 8]. The basic idea behind this reasoning process is that agents consider ex post whether they could have obtained a higher payoff by having made other choices and revise their choices in the direction of potentially higher payoffs. Or, as it is nicely explained by a metaphor that is close to our situation in [18], “Consider a marksman who repeatedly tries to hit the trunk of a tree with a bow and arrow. After a miss he will have the tendency to aim more to the left if the arrow passed the trunk to the right, and more to the right in the opposite case.”

**Fig 7:**
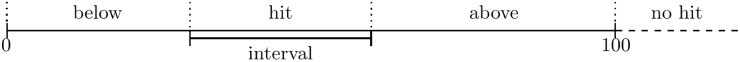
The four possible experiences.

We distinguish four mutually exclusive and jointly exhaustive experiences, depending on the termination time of the time series relative to the chosen interval: (1) the termination time is below the interval, (2) the termination time is in the interval, (3) the termination time is above the interval, but the time series terminated before *t* = 100, and (4) the time series did not terminate before *t* = 100. We label these possible experiences by ‘below’, ‘hit’, ‘above’, and ‘no hit’, respectively (see Fig 7). Only the experience ‘hit’ yields a positive payoff; the other experiences do not yield any payoff.

We use the following fixed effects regression model to estimate how individuals adapt their interval in response to these experiences:

$$\begin{array}{ccc} \Delta \ell_{i,t} & = & \alpha_{0}\;+\;\alpha_{1}\;{\text{Below}}_{i,t-1}\;+\;\alpha_{2}\;{\text{Above}}_{i,t-1}\;+\;\alpha_{3}\;{\text{NoHit}}_{i,t-1}\\ & & +\;\beta_{0}\;\text{2ndHalf}\;+\;\beta_{1}\;{\text{Below}}_{i,t-1}\;\times \;\text{2ndHalf}\;+\;\beta_{2}\;{\text{Above}}_{i,t-1}\;\times \;\text{2ndHalf}\\ & & +\;\beta_{3}\;{\text{NoHit}}_{i,t-1}\;\times \;\text{2ndHalf}\;+\;u_{i}\;+\;\varepsilon_{i,t}. \end{array}$$

Here, Δℓ_*i*,*t*_ denotes the change in either the location or the length of the interval of individual *i* in period *t*. The location of the interval refers to the mid-point of it. The results are shown in Table 4 and indicate that subjects react quite significantly to previous period experiences. The constant reported in the table is estimated under the constraint that the average *u*_*i*_ equals zero. The hypotheses at the bottom of the table test for the significance of the absolute effect of the different outcomes. For instance, since *α*_1_ captures the relative effect of ‘below’ to a hit (the omitted outcome), *H*_1_ (i.e. *α*_0_ + *α*_1_ = 0) tests whether there is a significant absolute effect of below in the first half of the experiment regardless of what happens when there is a hit. S2 Table shows similar specifications where the dependent variables represent changes in the lower and the upper bound instead.

**Table 4:** Interval updating depending on the experiences in the previous period.

	Low volatility			High volatility		
	Δlocation	Δlength	Δperf.	Δlocation	Δlength	Δperf.
Constant [*α*_0_]	0.5628 (3.1588)	-4.2195 ** (2.0100)	0.1003 ** (0.0470)	2.1735 (1.6217)	-3.7277 ** (1.5903)	0.0087 (0.0193)
Below (*t* − 1) [*α*_1_]	-31.5201 ** (14.2467)	15.6405 (12.3230)	-0.2123 (0.1300)	-11.5312 *** (2.7136)	7.7377 *** (2.2989)	0.0267 (0.0398)
Above (*t* − 1) [*α*_2_]	5.5832 (4.8707)	5.7291 (3.9233)	-0.0828 (0.0728)	21.0842 *** (4.9252)	14.0739 ** (6.4460)	-0.0165 (0.0737)
NoHit (*t* − 1) [*α*_3_]	0.4055 (3.8728)	4.9576 ** (2.2288)	-0.1013 * (0.0553)	-8.7336 *** (3.0723)	5.3295 ** (2.2856)	0.0053 (0.0296)
2ndHalf [*β*_0_]	1.5463 (3.8321)	2.0972 (2.4375)	-0.0633 (0.0552)	1.0374 (1.5720)	0.6579 (1.8760)	0.0056 (0.0225)
Below (*t* − 1) × 2ndHalf [*β*_1_]	20.4452 (13.7673)	-11.1367 (12.1676)	0.0915 (0.1425)	0.8516 (3.0497)	0.4224 (3.7297)	-0.0408 (0.0449)
Above (*t* − 1) × 2ndHalf [*β*_2_]	12.2142 (11.0755)	-12.3758 (7.5932)	0.0294 (0.0906)	-19.4786 *** (4.3711)	-10.6513 * (6.0427)	-0.0251 (0.0680)
NoHit (*t* − 1) × 2ndHalf [*β*_3_]	-2.4451 (4.5550)	-2.8978 (2.5647)	0.0698 (0.0603)	3.8284 (3.0954)	-1.0931 (2.7786)	-0.0155 (0.0360)
Fixed Effects	Y	Y	Y	Y	Y	Y
*F-test (p-values)*						
*H*_1_: (*α*_0_ + *α*_1_) = 0	0.0569	0.3577	0.4131	0.0000	0.0063	0.1928
*H*_2_: (*α*_0_ + *α*_2_) = 0	0.1274	0.6366	0.7532	0.0000	0.0680	0.9108
*H*_3_: (*α*_0_ + *α*_3_) = 0	0.3087	0.1474	0.9334	0.0007	0.1412	0.4526
*H*_4_: (*α*_0_ + *β*_0_) = 0	0.3385	0.1438	0.2323	0.0451	0.0063	0.3537
*H*_5_: (*α*_0_ + *β*_0_ + *α*_1_ + *β*_1_) = 0	0.0035	0.4700	0.1071	0.0126	0.0435	0.9964
*H*_6_: (*α*_0_ + *β*_0_ + *α*_2_ + *β*_2_) = 0	0.1262	0.2210	0.7371	0.0347	0.7710	0.1819
*H*_7_: (*α*_0_ + *β*_0_ + *α*_3_ + *β*_3_) = 0	0.8535	0.8407	0.3139	0.1678	0.4015	0.8131
Observations	684	684	684	684	684	684
R-squared	0.0540	0.0266	0.0265	0.1247	0.0524	0.0144

Standard errors clustered on the individual level in parentheses.

*** *p* < 0.01,

** *p* < 0.05,

* *p* < 0.1

After a successful ‘hit’ experience, individuals shrink their intervals in both treatments. This change in interval length is observed during the first half as well as during the second half of the experiment. While subjects on average increase the midpoint of the interval after this experience, this movement is only significant in the high volatility treatment during the second half of the experiment. In both treatments the movements induce an improvement in the expected payments, as measured by unconditional performance, in the following round, but only significantly so during the first half in the low volatility treatment. None of these dynamic responses are factual inconsistent with the directional learning paradigm.

When subjects experienced a termination below the selected interval in the previous period, they shift the interval downwards. Moreover, they increase the length of the interval, but this effect is only significant in the high volatility treatment. These dynamic responses are in accordance with the directional learning paradigm. The impact of these changes on expected payments are insignificant, but the direction (as identified by the coefficient) are opposite in the two treatments: in the low volatility treatment performance decreases while it increases in the high volatility treatment.

In case the series terminated above the chosen interval, in both treatments, individuals shift their intervals upwards; though, this change is only significant in the high volatility treatment. There is no notable effect on the chosen length of the interval. Again, the response to this unsuccessful experience is consistent with the principle of directional learning. As after the experience ‘below’, we also do not find a significant change in performance after the experience ‘above’.

In the more extreme case where the time series did not terminate before *t* = 100 (the ‘no hit’ experience), subjects increase the length of their chosen interval only significantly in the low volatility treatment during the first half of the experiment. While the midpoint of the interval moves slightly upwards (but this effect is insignificant) in the low volatility treatment, we find a significant movement downwards in the high volatility treatment but this reaction is insignificant in the second half of the experiment due to invariance of the upper bound (see *H*_7_ in S2 Table). While this reaction is not inconsistent with directional learning in the low volatility treatment, it is inconsistent with this learning paradigm in the high volatility treatment. Individuals seem prone to the gambler’s fallacy (cf. [19]) in the high volatility treatment by acting in accordance to the mistaken belief that, in order to balance the mean, a no hit should be followed by an early hit. Subjects do not manage to improve their performance significantly after the ‘no hit’ experience.

## Conclusion

In this paper we experimentally apply the interval scoring rule to elicit forecasts. In our experiment subjects have to forecast, over a sequence of twenty periods, the termination time of a time series that is to be generated from a fixed but unknown random process by specifying an interval where they believe the time series is going to terminate. We study the choices individuals make in this environment, how these choices change over time in response to recent experiences, how individual forecasting performance relates to the level of the underlying uncertainty and individual attributes like cognitive ability, risk attitude and gender.

We find that individuals report better predictions in the low volatility treatment compared to the high volatility treatment, and there is very little indication that this is due to any of the individual attributes elicited in our experiment. Over time individuals improve their forecasting performance in both treatments, although in the high volatility treatment they mainly improve the choice of location given the length but fail to choose the correct length. The quality of elicited beliefs may therefore be lower in highly uncertain environments. Although subjects learn by experience in a way mostly consistent with directional learning, there is evidence for subjects being prone to the gambler’s fallacy in the high volatility treatment. All in all, on the basis of individual choices, we can conclude that the amount of uncertainty has a large impact on individuals’ forecasts when using the interval scoring rule.

In contrast to our findings on the effect of environmental uncertainty, observed individual characteristics do not systematically affect the location and length of the elicited intervals. This shows the robustness of the interval scoring rule as an elicitation mechanism as long as proper incentives are provided.

## Supporting information

S1 Fig Observed innovations prior to the first round of decision making against the reference distribution. (PDF)

S2 Fig Simulated termination distributions from observed example series prior to first round of decision making against the reference distribution. (PDF)

S1 Table Remake of Table 2 with upper and lower bound. (PDF)

S2 Table Remake of Table 4 with upper and lower bound. (PDF)
