# Supplementary material for: Eliciting interval beliefs: An experimental study
Source: PLoS One. 2017 Apr 5;12(4):e0175163. doi: 10.1371/journal.pone.0175163 (PMC5381926; doi:10.1371/journal.pone.0175163)
Supplement: S1 Fig — (PDF) [file pone.0175163.s001.pdf]

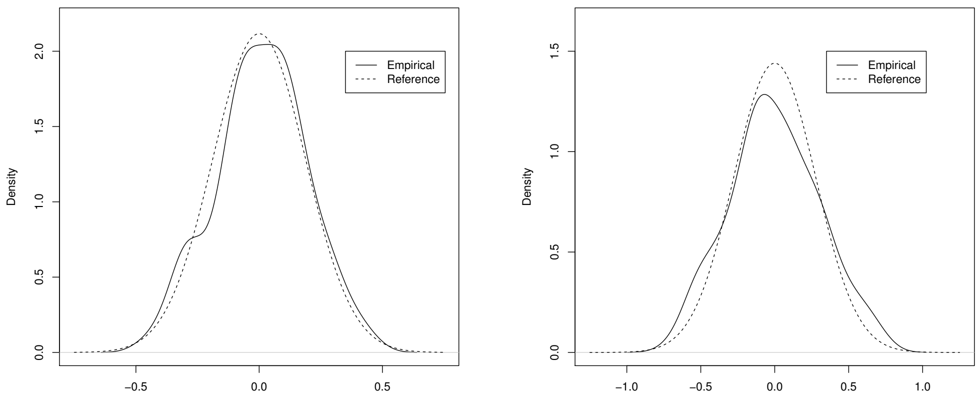


(a) Low volatility (b) High volatility

**S1 Fig. Observed innovations prior to the first round of decision making against the reference distribution.**
