# Supplementary material for: Eliciting interval beliefs: An experimental study
Source: PLoS One. 2017 Apr 5;12(4):e0175163. doi: 10.1371/journal.pone.0175163 (PMC5381926; doi:10.1371/journal.pone.0175163)
Supplement: S2 Fig — (PDF) [file pone.0175163.s002.pdf]

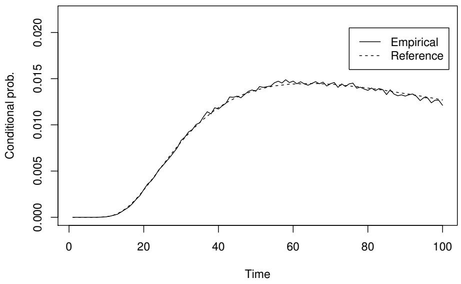

(a) Low volatility

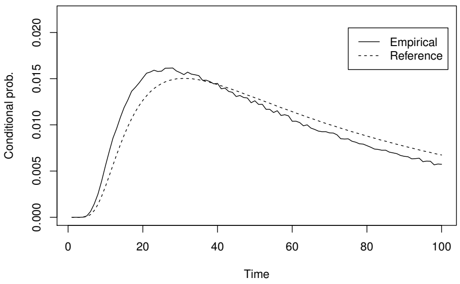

(b) High volatility

S2 Fig. Simulated termination distributions from observed example series prior to first round of decision making against the reference distribution.
