# Supplementary material for: Eliciting interval beliefs: An experimental study
Source: PLoS One. 2017 Apr 5;12(4):e0175163. doi: 10.1371/journal.pone.0175163 (PMC5381926; doi:10.1371/journal.pone.0175163)
Supplement: S1 Table — (PDF) [file pone.0175163.s003.pdf]

S1 Table. Remake of Table 2 with upper and lower bound.

	Low		High	
Treatment	Lower bound	Upper bound	Lower bound	Upper bound
Constant	95.5222**	150.1035***	121.1644**	−14.4597
	(36.9779)	(36.6531)	(54.8319)	(49.2476)
2nd Half	3.9972***	1.0361	−3.3194**	−0.6278
	(1.4328)	(1.0910)	(1.3259)	(1.3003)
Gender	1.8260	1.0198	−1.4144	5.0106
	(2.5694)	(3.5586)	(3.0035)	(3.7823)
Risk attitude	0.0282	−0.2190	−0.5080	−1.1297
	(0.9215)	(0.9144)	(0.7315)	(0.7020)
Cognitive ability	−2.8750	−3.4825*	−4.1900	4.9281*
	(1.7631)	(1.9527)	(2.7499)	(2.5702)
Cognitive ability	0.0359	0.0423*	0.0517	−0.0623*
(squared)	(0.0216)	(0.0246)	(0.0346)	(0.0335)
Observations	720	720	720	720
R-squared	0.0341	0.0195	0.0232	0.0378

Standard errors clustered on the individual level in parentheses.

$^{***}p < 0.01$, $^{**}p < 0.05$, $^{*}p < 0.1$.
