# Supplementary material for: Eliciting interval beliefs: An experimental study
Source: PLoS One. 2017 Apr 5;12(4):e0175163. doi: 10.1371/journal.pone.0175163 (PMC5381926; doi:10.1371/journal.pone.0175163)
Supplement: S2 Table — (PDF) [file pone.0175163.s004.pdf]

S2 Table. Remake of Table 4 with upper and lower bound.

	Low		High	
Treatment	Δ Lower bound	Δ Upper bound	Δ Lower bound	Δ Upper bound
Constant [α_0]	2.6725	-1.5470	4.0373^{**}	0.3096
	(3.3824)	(3.2458)	(1.8785)	(1.7308)
Below $(t-1)$ [α_1]	-39.3403^{***}	-23.6998	-15.4000^{***}	-7.6623^{***}
	(12.3101)	(18.1749)	(3.2273)	(2.6371)
Above $(t-1)$ [α_2]	2.7187	8.4478	14.0472^{**}	28.1211^{***}
	(4.7587)	(5.7008)	(5.3077)	(6.4124)
NoHit $(t-1)$ [α_3]	-2.0733	2.8843	-11.3984^{***}	-6.0689^{*}
	(4.1099)	(3.9484)	(3.3605)	(3.1933)
2ndHalf [β_0]	0.4977	2.5949	0.7084	1.3663
	(4.1672)	(3.8698)	(1.7755)	(1.8841)
Below $(t-1)$ × 2ndHalf [β_1]	26.0135^{**}	14.8768	0.6404	1.0628
	(12.0138)	(17.5719)	(3.9587)	(3.1442)
Above $(t-1)$ × 2ndHalf [β_2]	18.4021^{**}	6.0263	-14.1529^{**}	-24.8042^{***}
	(8.6233)	(14.1350)	(5.4855)	(5.1360)
NoHit $(t-1)$ × 2ndHalf [β_3]	-0.9962	-3.8940	4.3749	3.2818
	(4.8440)	(4.6175)	(2.8384)	(3.8687)
Fixed Effects	Y	Y	Y	Y
F-test (p-values)				
$H_1 : (\alpha_0 + \alpha_1) = 0$	0.0112	0.2049	0.0000	0.0011
$H_2 : (\alpha_0 + \alpha_2) = 0$	0.1588	0.1500	0.0005	0.0000
$H_3 : (\alpha_0 + \alpha_3) = 0$	0.5345	0.1831	0.0004	0.0030
$H_4 : (\alpha_0 + \beta_0) = 0$	0.1901	0.6364	0.0055	0.3201
$H_5 : (\alpha_0 + \beta_0 + \alpha_1 + \beta_1) = 0$	0.0061	0.0167	0.0069	0.0695
$H_6 : (\alpha_0 + \beta_0 + \alpha_2 + \beta_2) = 0$	0.0122	0.3452	0.0387	0.0433
$H_7 : (\alpha_0 + \beta_0 + \alpha_3 + \beta_3) = 0$	0.8098	0.9230	0.0468	0.4967
Observations	684	684	684	684
R-squared	0.0609	0.0393	0.1234	0.1053

Standard errors clustered on the individual level in parentheses.
$^{***}p < 0.01$, $^{**}p < 0.05$, $^{*}p < 0.1$
